# Low-dose Synachten test with measurement of salivary cortisol in adult patients with β-thalassemia major

**DOI:** 10.1007/s12020-018-1562-z

**Published:** 2018-03-23

**Authors:** Alberto G. Ambrogio, Leila Danesi, Marina Baldini, Raffaella Radin, Elena Cassinerio, Giovanna Graziadei, Nadia Mirra, Emanuela D’Angelo, Alessia Marcon, Marta Mancarella, Alessandra Orsatti, Federico Bonetti, Massimo Scacchi, Maria Domenica Cappellini, Luca Persani, Francesca Pecori Giraldi

**Affiliations:** 1 Neuroendocrine Research Laboratory, Istituto Auxologico Italiano IRCCS, Milan, Italy; 2 Division of Endocrine and Metabolic Diseases, Ospedale San Luca, Istituto Auxologico Italiano IRCCS, Milan, Italy; 3 Department of Medicine and Medical Specialties, Rare Diseases Center, Fondazione IRCCS Ca’ Granda Ospedale Maggiore Policlinico, Milan, Italy; 4 Fondazione Policlinico Mangiagalli Regina Elena, Second Pediatric Clinic, University of Milan, Milan, Italy; 5 Department of Clinical Sciences and Community Health, University of Milan, Milan, Italy; 6 Pediatric Haematology Oncology, Fondazione IRCCS Policlinico San Matteo, Pavia, Italy; 7 Division of General Medicine, Istituto Auxologico Italiano IRCCS, Ospedale San Giuseppe, Piancavallo-Verbania, Via San Vittore, Italy

**Keywords:** Adrenocortical insufficiency, β-thalassemia major, Salivary cortisol, Serum cortisol, Low-dose ACTH stimulation test, Cortisol-binding globulin

## Abstract

**Purpose:**

Beta-thalassemia major is a severe, congenital hematological disorder and, if untreated, leads to early mortality. Progress in therapeutical strategies improved clinical outcomes and life expectancy; however, increased survival led to the development of new disorders, including endocrinopathies. Little is known on the possible impairment of adrenocortical function, a potentially life-threatening condition, in long-term thalassaemic survivors. We therefore decided to assess adrenal reserve and the value of salivary cortisol during ACTH stimulation in the diagnosis of adrenocortical insufficiency in adult patients with β-thalassemia major.

**Methods:**

Cross-sectional study including 72 adults with β-thalassemia major. Patients were tested with 1 µg ACTH for serum and salivary cortisol.

**Results:**

Subnormal serum cortisol responses to ACTH stimulation (i.e., <500 nmol/l) were registered in 15 out of 72 patients. Salivary cortisol increased in parallel with serum cortisol and a clear-cut positive correlation was detected at each timepoint. Moreover, peak salivary cortisol values after ACTH stimulation were significantly lower in patients with impaired adrenal reserve (513.6 ± 52.33 vs. 914.1 ± 44.04 nmol/l *p* < 0.0001).

**Conclusions:**

Our results attest to the need for testing for adrenal insufficiency among adult thalassaemic patients, as up to 20% presented impaired adrenal reserve. Salivary and serum cortisol levels during stimulation with ACTH were closely correlated and the use of salivary cortisol sampling during ACTH testing may represent a surrogate to serum cortisol in these patients.

## Introduction

Beta-thalassemia major is a severe, congenital hematological disoder leading to ineffective erythropoiesis. Transfusion and chelation therapy have dramatically ameliorated life expectancy in these patients but increased survival concurrently led to the development of new complications [1]. Endocrine deficiencies rank among the most frequent ancillary disorders in ß-thalassemia major and most have been exhaustively studied and treated in young patients. However, the absence of consistent studies in adult thalassaemic patients may lead to an underestimation of the impact of endocrine disorders once puberty and growth have been completed.

Hemosiderosis is the main cause of endocrine tissue damage in ß-thalassemia major [2]. In fact, saturation of serum transferrin binding capacity leads to accumulation of free iron, generation of reactive oxygen species and increased iron uptake by organs with high transferrin receptor density, such as liver, heart, and the endocrine glands [3]. Other causes of endocrine tissue injury are hypoxia [4], secondary to chronic anemia, and liver disease, the latter caused by iron overload or transfusion-related viral hepatitis [5].

Adrenal insufficiency is an insidious, protean condition which may prove fatal if untreated. Failure to diagnose this condition may have serious consequences during major stress events such as surgery, trauma or severe infections, as the adrenal will be unable to mount an adequate response [6]. Moreover, main symptoms and signs of adrenal insufficiency, i.e., fatigue, anorexia, weight loss and depression, are non-specific and may easily be confused with symptoms due to chronic anemia. As the diagnosis of adrenocortical hypofunction rests on specific laboratory assessments [7], it has to be suspected and explicitly sought for.

Currently available literature on adrenal insufficiency in ß-thalassemia major pertains almost exclusively to children or adolescents and the reported prevalence of adrenocortical failure appears extremely variable, ranging from 0% to over 80%, according to the degree of iron overload and the diagnostic criterion used [8, 9]. On the other hand, little is known to date on adrenocortical function in adult subjects with ß-thalassemia major. We have previously assessed adrenal reserve by low and high dose ACTH stimulation test in adults with ß-thalassemia major and observed approximately 30% subnormal responses [10].

Aims of the present study were (a) to estimate the prevalence of adrenocortical insufficiency as assessed with low dose ACTH stimulation a large series of adult thalassaemic patients; (b) to explore the usefulness of salivary cortisol during ACTH testing for the diagnosis of adrenocortical insufficiency; (c) to establish the impact of this endocrinopathy on the quality of life of patients with ß-thalassemia major.

## Patients and methods

### Patients

We recruited 72 consecutive adult patients (44 women, 28 men; age 40.1 ± 0.88 years, range 24–60) with ß-thalassemia major over 2 years. Only patients started on blood transfusions and iron chelation therapy within a few months from birth were included. Defects in gonadal and thyroid function, if present, were corrected using testosterone injections or estrogen plus progesterone medication and oral thyroid supplementation and all patients were euthyroid at current evaluation. Patients with pre-transfusion hemoglobin levels below 7 g/dl, concurrent acute events, severe hepatic disease were excluded. None of the patients had been previously tested [10] or treated for adrenal insufficiency. Further, no patient had undergone serious clinical events or been treated with systemic corticosteroids. The study protocol was approved by the Ethical Committee of IRCCS Istituto Auxologico Italiano and Fondazione IRCCS Ca’ Granda Ospedale Maggiore Policlinico and written informed consent obtained from all participants prior to testing.

### Study design

Blood samples for measurement of cortisol, ACTH, prolactin, TSH, and free T4 concentrations were collected between 8 and 9 AM. Standard clinical chemistry testing and estimation of ferritin levels were also performed.

Adrenocortical reserve was assessed by low-dose ACTH testing (1 μg Synacthen, Sigma-Tau Industrie Riunite, Rome, Italy). Blood and saliva samples were collected prior to testing and 15, 30, 60, and 90 min after ACTH i.v. bolus. Saliva was collected into Salivette swabs (Sarstedt AG & Co., Nümbrecht, Germany). Peak serum cortisol levels below 500 nmol/l (18 μg/dl) were considered indicative of reduced adrenocortical reserve [11].

Quality of life (QoL) was established by Short Form-36 Health Survey (SF-36). The questionnaire has been validated by standard methodology of International Quality of Life Assessment [12].

### Biochemical assays

All hormone assays were performed at the Istituto Auxologico Italiano. Concentrations in serum and urine were measured at by electrochemiluminescence (Roche Diagnostics, Mannheim, Germany). Sensitivity of assays was 0.22 pmol/l for ACTH and 0.5 nmol/l for serum and salivary cortisol; intra-assay and inter-assay coefficient of variations were 2.9 and 5.4%, 1.7 and 2.2%, and 2.8 and 4.1% for ACTH, serum and salivary cortisol, respectively.

### Imaging

Liver and cardiac MRI were performed at CMR Unit Department of Cardiology “A. De Gasperis” at Niguarda Ca’ Granda Hospital in Milan, using a 1.5 T MR scanner (Avanto Siemens, Erlangen, Germany). All T2* images were analyzed using post-processing software (CMR Tools, Imperial College, London). CMR assessment was performed by a single operator blinded to patients’ clinical data. Parameters for assessment of cardiac siderosis by T2* were: normal >20 ms; moderate-to-mild between 10 and 20 ms and severe <10 ms; for liver siderosis: normal >6.3 ms; moderate-to-mild between 1.4–6.3 ms and severe <1.4 ms. Liver iron concentration (LIC) was calculated from liver T2* by the formula [1/(T2*/1000)] x 0.0254 + 0.202 [13].

### Statistical analysis

Statistical analysis was performed using commercially available software (StatView, Abacus Concepts, Berkeley CA, USA and MedCalc Software, Ostend, Belgium). Data are expressed as mean ± standard error of the mean (SE). Baseline ACTH, cortisol and prolactin levels were averaged over two samples collected 20 min apart. Mann–Whitney test for unpaired data was used for comparisons between groups and linear regression analysis for associations between variables. Chi-test statistics was used for assessment of qualitative variables. Receiver operator characteristic (ROC) analysis was performed to assess the diagnostic efficiency of salivary cortisol and calculate Youden’s J index [14]. Logistic regression analysis was used for assessment of factors predictive of impaired adrenal reserve. *P*-values <0.05 were considered statistically significant.

## Results

Demographic, biochemical and hormonal data in patients are shown in Table 1. As can be observed, patients presented normal liver function, adequate hemoglobin levels, and moderately high ferritin levels, as expected in regularly transfused, well-chelated thalassaemic patients.

**Table 1:** Demographic and biochemical data in patients with thalassemia major

	Mean ± SEM	Reference values
BMI (kg/sm)	23 ± 0.39	18–25
Hemoglobin (mmol/l)	6.14 ± 0.08	7.45–11.17
Albumin (g/l)	42.7 ± 0.63	32–52
AST (U/l)	31 ± 3.00	<40
ALT (U/l)	34 ± 4.50	<40
Creatinine (µmol/l)	64.41 ± 2.36	35–132
Sodium (mmol/l)	140 ± 0.34	136–147
Potassium (mmol/l)	4 ± 0.05	3.5–5.4
Ferritin (pmol/l)	2755.6 ± 432.43	67–900
TSH (mU/l)	3 ± 0.18	0.27–4.5
FT4 (pmol/l)	17 ± 0.45	11.5–24.5
Prolactin (μg/l)	M: 11.5 ± 1.59 F: 8.9 ± 0.71	M: 2.5–17 F. 3–20
ACTH (pmol/l)	4.25 ± 0.31	2.2–11
Serum cortisol (nmol/l)	347.4 ± 20.41	140–680
LIC (Fe/g crude weight)	5 ± 0.56	Normal: <3
		Slight iron overload: 3–7
		Moderate iron overload: 7–14
		Severe iron overload: >14
Liver T2* (ms)	10 ± 1.00	Normal: >6.3
		Slight iron overload: 2.6–6.3
		Moderate iron overload: 1.4–2.6
		Severe iron overload: <1.4
Cardiac T2* (ms)	35 ± 1.70	Normal: >20
		Slight iron overload: 14–20
		Moderate iron overload: 10–14
		Severe iron overload: <10

*F* females, *M* males, *SEM* standard error of the mean

Basal serum cortisol levels were mostly comprised in the reference range and only two patients presented values below the lower limit of normal. No patient presented serum cortisol below 80 nmol/l (3 µg/dl).

An impaired serum cortisol response to ACTH was observed in 15 patients (21%). On average, patients with impaired adrenal reserve presented higher ACTH values compared to those with normal adrenal reserve (5.51 ± 0.81 vs. 3.92 ± 0.31 pmol/l, *P* < 0.05) and, indeed, peak cortisol levels were negatively correlated with plasma ACTH concentrations (*r* = −0.293, *P* < 0.05). Three patients, all with impaired adrenal reserve, presented supranormal ACTH concentrations, i.e., >11 pmol/l. Obviously, basal serum cortisol levels (247.1 ± 15.58 vs. 373.4 ± 24.34 nmol/l, *P* < 0.01) were reduced in the patients with impaired adrenal reserve. Thalassaemic men presented lower peak cortisol values compared to their female counterparts (16509.8 ± 889.5 vs. 19793.8 ± 1010.3 nmol/l, *P* < 0.05) but comparable adrenal reserve status (impaired in 25 vs. 18% men and women, NS), as peaks were clearly above the established cut-off, i.e., 500 nmol/l (18 µg/dl), in both groups. No differences in terms of age (38.3 ± 1.62 vs. 40.6 ± 1.02 years, NS), sodium (140.2 ± 0.55 vs. 139.8 ± 0.34 mmol/l, NS) or blood glucose (4.9 ± 0.19 vs. 5.8 ± 0.36 mmol/l, NS) levels were observed between patients with impaired or preserved adrenal function.

**Fig. 1:**
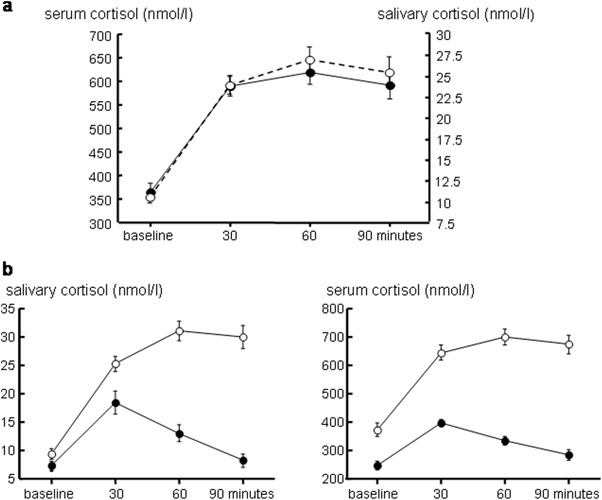
Serum and salivary cortisol response to ACTH testing (mean ± SEM) **a** Response in the entire series. Filled circles indicate serum cortisol, empty circles and dashed line indicate salivary cortisol. **b** serum and salivary cortisol values during ACTH testing in patients with impaired (filled circles) or preserved (empty circles) adrenal reserve

Salivary cortisol concentrations increased in parallel with serum cortisol after ACTH stimulation (Fig. 1a) and a significant correlation between serum and salivary cortisol was detected at baseline (*r* = 0.281, *P* < 0.05), peak (*r* = 0.494, *P* < 0.005) and all test times (30 min: *r* = 0.360, *P* < 0.005; 60 min: 0.524, *P* < 0.001; 90 min: *r* = 0.678, *P* < 0.001). Baseline and peak salivary cortisol levels were comparable among sexes (baseline: 8.8 ± 1.04 vs. 9.2 ± 1.18 nmol/l, N.S:, peak: 755.3 ± 39.8 vs. 932.2 ± 80.4 nmol/l, N.S. for women and men, respectively).

**Fig. 2:**
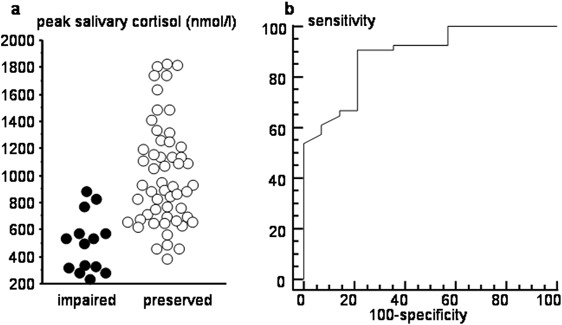
Salivary cortisol response to ACTH testing. **a** Peak salivary cortisol values at ACTH testing in patients with impaired (filled circles) or preserved (empty circles) adrenal reserve. **b** ROC curve for peak salivary cortisol at ACTH testing

For cortisol in both fluids, clear differences in the response to low-dose ACTH between patients with impaired or preserved adrenal reserve could be observed (Fig. 1b). Indeed, peak salivary cortisol was significantly lower in the patients with impaired adrenal reserve (513.6 ± 52.33 vs. 914.1 ± 44.04 nmol/l *P* < 0.0001; Fig. 2a). ROC analysis showed that peak salivary cortisol levels higher than 21.8 nmol/l discriminated, with good diagnostic accuracy (Youden J index 0.707, 95% confidence interval 0.518; 0.891; Fig. 2b), between patients with adrenocortical insufficiency and those with normal adrenal reserve.

**Fig. 3:**
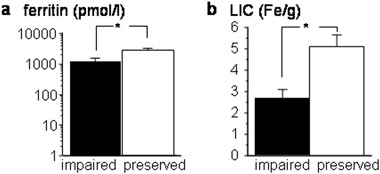
Iron overload parameters and adrenal reserve testing (mean ± SEM). Differences in ferritin (**a**) and LIC (**b**) according to adrenal reserve status, *P = or < 0.05

Ferritin levels were slightly higher in patients with normal adrenal reserve compared to patients with impaired adrenal function (3085.6 ± 517.8 vs. 1309.4 ± 298.2 pmol/l, respectively, *P* = 0.05; Fig. 3a) as was LIC (5.08 ± 0.575 vs. 2.68 ± 1.294 ms, respectively, *P* < 0.05; Fig. 3b); indeed, peak serum cortisol was correlated with LIC and liver T2* (*r* = 0.322, *P* < 0.005 and *r* = −0.292, *P* < 0.05, respectively). Cardiac T2* did not differ among patients with preserved or impaired adrenal reserve (34.7 ± 1.87 vs. 36.1 ± 2.61, N.S:, respectively). Hemoglobin levels were comparable between patients with impaired or preserved adrenal reserve (5.94 ± 0.159 vs. 6.18 ± 0.096 mmol/l, N.S.) as was the time interval between transfusions (22.0 ± 2.06 vs. 22.3 ± 1.25 days, NS). At logistic regression analysis, only LIC was retained as a significant predictor of cortisol response status (odds ratio 0.682, 95% confidence interval: 0.466, 0.992).

Lastly, quality of life was impaired in the physical and mental health domains (PCS standardized score −0.292 ± 0.112, MCS −0.215 ± 0.136) in thalassaemic patients per se. No difference in SF-36 scores, both for individual domains and cumulative scores, was observed between patients with impaired or preserved adrenal reserve (PCS −0.069 ± 0.194 vs. −0.340 ± 0.0129 and MCS −0.008 ± 0.220 vs. −0.259 ± 0.158 respectively, both NS).

## Discussion

Life expectancy in patients with β-thalassemia major has increased considerably thanks to newer and better transfusion and chelation regimens. However, this improvement is not scot-free as patients with ß-thalassemia major may develop a variety of ailments from cardiac diseases to endocrinopathies, indeed, the latter rank among the most prevalent [15]. Endocrine disorders have been extensively studied in children and adolescents, in whom growth failure and delayed puberty are common [16] whereas only few studies investigated endocrine function in adults with ß-thalassemia major. Over the past decade, we have attempted to fill this gap and reported reduced bone mineral density [17], severe growth hormone (GH) deficiency [18] and subtle alterations in adrenal function [10] in adult thalassaemic patients. The latter finding led to the present study, the largest series of adult patients with β-thalassemia major tested for adrenal reserve to date, which allowed us to obtain solid data on the prevalence of adrenal insufficiency in adult thalassemic patients and propose salivary cortisol as a substitute marker for cortisol response during Synachten testing.

Our results show that 20% of adult thalassaemic patients present reduced adrenocortical reserve, as assessed by low-dose ACTH testing. This result is in line with our previous study using using low-dose and high-dose ACTH [10]. Conversely, another study on 25 adult patients tested with glucagon reported abnormal reserve in one patient [19]; however, the cut-off used to establish adrenal insufficiency was set at 599 nmol/l (21 µg/dl), thus some patients may have been missed.

It is worth recalling that the diagnosis of adrenal insufficiency rests on the measurement of serum cortisol at baseline and after stimulation [7, 11]. Two studies dating back to the 1990s had used insulin-induced hypoglycemia [8, 20], the gold standard for adrenal insufficiency [21], to test adrenal reserve in ß-thalassemia major but this test should be avoided in multi-complicated patients [22]. In children with ß-thalassemia major, tests and criteria used to assess adrenal reserve varied considerably as both low-dose and high-dose ACTH and glucagon were used and cortisol response criteria ranged from 400 to 550 nmol/l (15–20 µg/dl) [9]. Further, most series reported small numbers of patients, 50 at most, thus it comes at no surprise that estimates of adrenal insufficiency in young patients varied from 15 to 87%. A more recent study on 120 children settled the debate at 32% prevalence [23], an estimate which does not differ considerably from the prevalence we observed in adult patients, suggesting that disease duration does not affect adrenocortical function. Indeed, in this as well as in our previous study [10], impairment of adrenal reserve was not correlated with age. Of note, since patients with impaired adrenal reserve presented higher ACTH values compared to patients with normal adrenal reserve and peak cortisol levels correlated negatively with plasma ACTH concentrations, the adrenal may be the primary cause of hypocortisolism in thalassaemic patients [10, 20].

As regards pathogenetic mechanisms, iron overload and/or chronic hypoxia have been proposed as the main determinants of endocrine complications in ß-thalassemia major [4]. Interestingly, impaired adrenal function was associated with less iron overload, as evaluated by serum ferritin levels and hepatic parameters. This finding has also been described in other, smaller series [9, 24] and could probably be explained by differences in tissue-specific iron deposition [25]. LIC levels were normal or slightly increased in the majority of the patients in keeping with adequate liver iron chelation. Unfortunately, we could not perform pituitary or adrenal MRI in order to evaluate iron overload of tissues involved in adrenal insufficiency. On the other hand, chronic hypoxia does not seem a likely contributor to adrenal dysfunction as hemoglobin levels and the time interval between transfusions proved comparable in patients with impaired and preserved adrenal reserve. Overall, the causes of adrenal insufficiency in thalassaemic patients remain to be established.

All tests performed during the diagnostic work-up of adrenocortical insufficiency require evaluation of serum cortisol levels, which may be influenced by variations in cortisol-binding globulin (CBG). It is worth recalling that CBG synthesis may be affected in patients with severe liver disease [26] and hepatic damage is a possible complication of ß-thalassemia major due to haemochromatosis and/or post-transfusion viral infections [27]. A study on some 50 patients with ß-thalassemia major [28], however, reported normal CBG levels. In any case, CBG-related issues may be overcome by measurement of salivary cortisol, which reflects the free cortisol fraction and is not influenced by CBG concentrations. Salivary cortisol has been already studied as a viable alternative to serum cortisol in the diagnosis of adrenocortical insufficiency [29], in particular in patients with liver disease [26]. We therefore decided to investigate the salivary cortisol response to ACTH stimulation in thalassaemic patients and can report that salivary cortisol rises in parallel with its serum counterpart, as documented by the close correlation between the two parameters. Indeed, ROC curve analysis of the salivary cortisol peak yielded good diagnostic accuracy and suggested a possible cut-off, to be validated by more extensive testing.

Our study confirmed low quality of life in adult thalassaemic patients, both in terms of physical and mental health scores [30]. This finding is not unexpected and due to several factors, including long duration of the disease, continued treatment regimens and related complications [31]. Not surprisingly, given the degree of quality of life impairment in ß-thalassemia per se, adrenocortical insufficiency did not further impact health survey estimates. On the other hand, it is worth recalling that adrenocortical insufficiency appeared mild in our patients, as none presented very low cortisol levels, i.e., <80 nmol/l (<3 µg/dl), or severe electrolyte or glycemic derangements, thus, subtle complaints are likely to be underappreciated in this context. In fact, the absence of clear-cut symptoms of adrenal insufficiency may lead the clinician to underestimate adrenal deficiency, with -possibly- fatal consequences in stressful conditions [6]. Patients diagnosed with impaired adrenal reserve in this series were started on steroid replacement therapy and advised on glucocorticoid stress-dosing; follow-up on these patients is ongoing and will hopefully yield interesting data in the near future.

Our study, as with all cross-sectional studies, is subject to some limitations. We tested each patient just once, thus cannot establish whether prevalence of adrenal insufficiency is progressive with length of disease. The absence of correlation with age argues against this hypothesis and retesting at later time-intervals may provide a definite answer to this question. As regards the impact of transfusion and chelation regimens given that patients were followed by different physicians, treatment had been started early in life in all patients and, indeed, hematologic parameters were indicative of well-chelated, regularly transfused patients.

In conclusion, the present study showed that a significant proportion of adult thalassaemic patients presents adrenocortical insufficiency in the largest series reported to date. It follows that adrenocortical function should be assessed in these patients in order to obtain a timely diagnosis and provide proper treatments. Salivary cortisol during ACTH stimulation may represent a valid, alternative measure of adrenocortical reserve, thus avoiding repeated blood sampling and increased disease burden.
